# Association of plasma zinc and copper levels with mild cognitive impairment in patients with type 2 diabetes

**DOI:** 10.3389/fnut.2025.1532080

**Published:** 2025-03-12

**Authors:** Yang Jiao, Xing Zhang, Lian Duan, Ruijie Cheng, Ning Yang, Zhao Peng, Ben Li, Lu Xu, Wenwen Chen, Jingrong Chen, Yanchao Liu, Hong Yan

**Affiliations:** ^1^Department of Health Toxicology, MOE Key Lab of Environment and Health, School of Public Health, Tongji Medical College, Huazhong University of Science and Technology, Wuhan, China; ^2^Department of Nutrition and Food Hygiene, Hubei Key Laboratory of Food Nutrition and Safety, School of Public Health, Tongji Medical College, Huazhong University of Science and Technology, Wuhan, China; ^3^Changsha Institute for Food and Drug Control, Changsha, China; ^4^Xiangyang Public Inspection and Testing Center, Xiangyang, China; ^5^Department of Neurosurgery, Tongji Hospital, Tongji Medical College, Huazhong University of Science and Technology, Wuhan, China

**Keywords:** type 2 diabetes mellitus, zinc, copper, mild cognitive impairment, case-control study

## Abstract

**Background:**

Type 2 diabetes mellitus (T2DM) is a significant risk factor for cognitive impairment. Zinc deficiency contributes to T2DM development, while copper may exacerbate diabetes through prooxidant mechanisms. Higher zinc levels may protect against copper toxicity. This study investigates the association of plasma zinc and copper levels with mild cognitive impairment (MCI) in T2DM patients.

**Methods:**

T2DM patients admitted to Tongji Hospital from 2012 to 2018 were classified into MCI (*n* = 136) and control (*n* = 136) groups, matched by age (± 3 years) and gender. Conditional logistic regression was used to assess the associations between plasma zinc, copper levels and MCI. A generalized additive model (GAM) evaluated the dose–response relationship between plasma zinc, copper levels and Mini-Mental State Examination (MMSE) scores.

**Results:**

The median of plasma metal levels in MCI and control groups were 831.31 μg/L and 936.29 μg/L for zinc, 932.07 μg/L and 860.47 μg/L for copper, and 0.91 and 1.11 for the zinc-to-copper (Zn/Cu) ratio. Compared to participants in the lowest tertile, the multivariable-adjusted odds ratios with 95% confidence intervals (CI) for MCI in the highest tertile were 0.33 (0.13, 0.79) for zinc, 3.56 (1.42, 8.94) for copper, and 0.37 (0.15, 0.93) for the Zn/Cu ratio. Plasma Aβ40 levels were significantly lower (*p* = 0.009) and plasma Aβ42/40 levels were significantly higher (*p* = 0.008) in MCI group compared with those in control group. Zinc concentration was positively associated with Aβ42. For per SD (327.71 μg/L) increase in plasma zinc levels, the percent change (95% CI) of Aβ42 were 2.90 (0.85, 4.99).

**Conclusion:**

Higher plasma zinc levels and higher Zn/Cu ratio were associated with lower odds of MCI in T2DM patients, while higher copper levels increased the risk of MCI. This study provides insights on plasma zinc, copper, and Zn/Cu ratio and Aβ of MCI, further studies are needed to clarify the underlying mechanisms for novel therapies that could prevent or cure multiple T2DM-related cognitive impairments.

## 1 Introduction

In recent years, type 2 diabetes mellitus (T2DM) has been identified as a major health concern, and it has been recognized as a significant risk factor for cognitive impairment (1). Recent data show that about 116 million people in China have diabetes, the highest in the world. Epidemiological studies have shown an increased risk of dementia among patients with diabetes (2). A recent meta-analysis estimated that about 45% of patients with T2DM experience mild cognitive impairment (MCI), which is also considered a complication of T2DM (3). Over the past two decades, studies have reported that the prevalence of MCI among older adults in China has ranged from 5 to 28% (4, 5). MCI is a transitional stage of cognitive impairment that is characterized by intermediate stage between normal aging and dementia (6). It is characterized by mild memory or cognitive impairment that does not meet the dementia diagnostic criteria. MCI can further develop into Alzheimer’s disease (AD), a progressive deterioration of the central nervous system (7). Individuals with diabetes have been shown to have a 1.5–2.0 times higher risk of developing cognitive decline, cognitive impairment, or dementia compared to those without diabetes (8). The prevalence of dementia and cognitive impairment among people with diabetes has been reported to be 13.1% for those aged 65–74 years and 24.2% for those aged 75 years and over (9). However, the exact pathophysiology of MCI in patients with diabetes remains unclear. Although many studies indicate an increased risk of developing dementia in diabetic populations, the association between T2DM and MCI, and its underlying mechanisms, requires further investigation.

Zinc and copper are essential trace elements that play a critical role in inflammation, oxidative stress, energy metabolism, and insulin homeostasis and have been implicated in the pathogenesis of T2DM (10, 11). Some studies have investigated the association of plasma zinc and copper levels with T2DM patients; however, the findings remain inconclusive. A cross-sectional study reported that in comparison with controls, serum zinc levels were lower in individuals with T2DM (12). Similarly, another study also showed that serum zinc levels were significantly reduced in patients with T2DM, with levels three times less than those in the control group (13). Conversely, a study suggested a possible relationship between increasing levels of zinc and the prevalence of T2DM (14). Meanwhile, research on copper levels in patients with T2DM remains controversial. A study had found that copper levels were elevated in diabetes mellitus patients (15). In contrast, another study found that serum copper levels were significantly lower in patients with T2DM compared to controls (16). The role of zinc, copper and particularly zinc-to-copper ratio (Zn/Cu) has gained increasing attention in recent years as markers for diabetes diagnosis and management (13). Moreover, the dyshomeostasis of trace metals, such as zinc and copper, has been associated with a decline in cognitive performance (17). Zinc homeostasis imbalance leads to reactive oxygen species (ROS) accumulation and the activation of neuronal apoptosis, contributing to cognitive decline and the formation of neurodegenerative diseases such as AD and MCI (18). It was demonstrated that copper dysregulation has been observed to affect 50–60% of patients with MCI or AD (19), while the accumulation of copper in the blood has been associated with dementia (20). Previous epidemiological studies have suggested that lower serum zinc and higher serum copper levels may be associated with impaired cognitive function (21). Nevertheless, studies investigating the association between plasma zinc and copper levels and MCI in patients with T2DM are limited.

In addition, the mechanisms underlying the progression of T2DM to MCI remain unclear. *β*-Amyloid polypeptide (β-Amyloid, Aβ), consisting of 36–43 amino acids, is the main component of amyloid plaques in the brain (22). Aβ can be conveyed from the brain into the bloodstream via several pathways, and about 40–60% of brain-derived Aβ is cleared in the periphery (23). T2DM patients exhibit higher plasma Aβ levels than healthy population (24). Primarily studies focused on investigating plasma Aβ as a potential biomarker for AD. The findings of these studies indicated that plasma Aβ40 and Aβ42 concentrations were higher in cognitively cases who eventually developed AD compared to the control group (25). Zinc is an essential component of several Aβ-lowering enzymes, which plays an important part in peripheral Aβ clearance and cognitive dysfunction development (10). It has been suggested that zinc deficiency in T2DM patients may impair Aβ clearance and AD development (26). Copper was shown to contribute to cognitive impairment pathogenesis through pro-oxidant properties (13), higher levels of zinc may protect against copper toxicity by competing for the same binding sites (27). However, no studies have investigated the association between plasma zinc levels, peripheral Aβ levels and cognitive function in T2DM.

Therefore, it is necessary to investigate the association between plasma zinc, copper, and Aβ levels with MCI in T2DM patients. This study aims to explore the roles of zinc and copper in T2DM-induced cognitive dysfunction and provide novel insights for the early AD prevention in T2DM patients.

## 2 Materials and methods

### 2.1 Study population

Participants in this study were T2DM patients who attended Tongji Hospital of Huazhong University of Science and Technology between 2012 and 2018 with the following inclusion criteria: (1) age ≥ 50 years; (2) able to complete cognitive function tests; (3) no history of severe head trauma; (4) no history of alcohol addiction; and (5) no history of psychiatric disorders. All participants completed the Mini-Mental State Examination (MMSE) instruction of trained professional investigators and were classified into two groups: T2DM with MCI (MCI group) and T2DM without MCI (Control group), based on MMSE scores. According to the guidelines of the National Alzheimer’s Coordinating Center, MCI was defined as MMSE score ≤ 26 (28, 29). Sample size estimation was performed based on the 1:1 matched case-control study equation:


$$N=\frac{{\left[Z_{\alpha }\sqrt{2\bar{p}\left(1-\bar{p}\right)}+Z_{\beta }\sqrt{p_{1}\left(1-p_{1}\right)+p_{2}\left(1-p_{2}\right)}\right]}^{2}}{{\left(p_{1}-p_{2}\right)}^{2}}$$

The following values were used: the odds ratio (OR) was 2; power of 95% and a reference seroprevalence of 30% for controls. Thus, the minimum sample size for either cases or controls was 127. The MCI and control groups were 1:1 matched by age (± 3 years) and gender, resulting in a final sample of 136 MCI and 136 controls. The study was reviewed and approved by the Ethics Committee of Tongji Medical College, Huazhong University of Science and Technology, and all participants signed the informed consent (Clinical trials. gov; Number: NCT01830998).

### 2.2 Blood sample collection and APOE genotype determination

After a 12-h fast, venous blood was collected in heparin tubes, centrifuged for 5 min (3,000 rpm, 4°C), and plasma, leukocytes, and red blood cells were separated, numbered, and stored at −80°C (30). The APOE genotype was detected by polymerase chain reaction (PCR) using a multiplex amplification-blocked mutation system. The primers were designed and synthesized for APOE with specific Cys primers (Cys112/ Cys158) and Arg primers (Arg112/ Arg158), and a common reverse primer (30–32).

### 2.3 Plasma zinc and copper level testing

Plasma metal concentrations were detected by inductively coupled plasma mass spectrometry (ICP-MS). Plasma samples were thawed at 4°C, vortexed and mixed. Then, 2,340 μL of sample diluent was added to a 5 mL lyophilized tube, followed by 60 μL of the sample. The mixture was shaken and mixed for 1 min, then sonicated for 10 min and centrifuged at 6000 rpm for 6 min. The detection limit of zinc and copper was 0.0199 μg/L and 0.0106 μg/L, respectively. In establishing the limit of quantitation, the concentration of the lowest standard solution (0.1 μg/L) was considered as the reference point. In order to guarantee the quality of the results, the human plasma certified reference material metal controls (ClinChek No. 8885) were analyzed for every 20 samples (33). The recovery values ranged from 80 to 120%. Furthermore, the relative standard deviation of the measured triplicate samples was within 5%, thereby ensuring the accuracy and precision of the experimental methods (34).

### 2.4 Plasma Aβ level testing

In this study, plasma Aβ40 (EEL-H0542c 90) and Aβ42 (E-EL-H0543c) levels were quantified using enzyme-linked immunosorbent assay (ELISA) kits. All procedures followed the manufacturer’s instructions. Briefly, blank samples, the Aβ40 and Aβ42 standards, or samples of this study (100 μL each) were added to coated 96-well plates and incubated for 90 min at 37°C. Following the decanting without washing, 100 μL of the biotinylated detection antibody working solution was added, and the plate was incubated at 37°C for 1 h. Following another decant, 350 μL of washing buffer was added to each well, and the plate was washed three times. Horseradish peroxidase-conjugated solution (100 μL each) was added and incubated for 30 min at 37°C. After five washes, 90 μL of substrate reagent was added, and the plate was incubated in the dark for 15 min at 37°C. Finally, 100 μL of stop solution was added, and the optical density (OD value) was recorded at 450 nm using a microplate reader (35).

### 2.5 Statistical analysis

Demographic data and biochemical indicators were presented by case and control. Continuous variables of baseline participant characteristics that were normally distributed were expressed as means (standard deviation). Continuous variables that were not normally distributed were expressed as medians (interquartile range). Categorical variables were expressed as frequencies (percentages). To compare the differences between the case and control groups, the Student’s t-test, Mann–Whitney U rank sum test, and chi-square test were used.

Conditional logistic regression models were used to estimate ORs and 95% CIs associated with MCI in T2DM patients. Plasma zinc, copper, and Zn/Cu levels were categorized into tertiles according to the distribution in control participants. Subsequently, several potential confounding variables were gradually adjusted in the following models. Model 1 was adjusted for sex (female or male), age (years), diabetes duration (years), and APOE ε4 carrying status (yes or no). Model 2 was additionally adjusted for BMI (<24, 24–28, or ≥ 28 kg/m^2^), family history of diabetes (yes or no), hypertension (yes or no), hyperlipidemia (yes or no), CHD (yes or no), current smoking status (yes or no), current drinking status (yes or no), and HbA1c level (<6.5%, or ≥ 6.5%).

To further investigate the dose–response relationship between metal exposure and the incidence of MCI in T2DM, a restricted cubic spline (RCS) regression analysis was conducted using the R package “rms.” In order to balance the optimal fitting and overfitting of the principal spline, the number of knots was set to three based on the minimum absolute value of the Akaike information criterion (36, 37). Finally, three knots corresponding to the 10th, 50th, 90th percentiles were used. The generalized additive model (GAM) was used to assess the non-linear relationship of plasma zinc and copper levels with MMSE score, as well as Aβ40 and Aβ42 levels. For the GAM analysis, Aβ40 and Aβ42 were log-transformed. In addition, β-coefficients were transformed into percent difference using the following formula: $\left(\exp^{\beta }-1\right)\times 100\%$ , to aid in the interpretation. Lastly, exposure-response curves were plotted based on the GAM.

All analyses in this study were performed using SPSS 26.0, R software (version 4.4.0), and SAS 9.4 software. All *p*-values were tested using a two-sided test, and *p* < 0.05 indicated statistical significance.

## 3 Results

### 3.1 Basic characteristics of the research subjects

The demographic data and clinical and biochemical indices of 136 cases and 136 matched controls are shown in Table 1. The medians (interquartile range) of plasma metal levels in MCI and control groups were 831.31 (717.91–1043.41) μg/L and 936.29 (827.41–1205.58) μg/L for zinc, 932.07 (808.73–1032.12) μg/L and 860.47 (750.25–974.34) μg/L for copper, and 0.91 (0.75–1.17) and 1.11 (0.91–1.34) for Zn/Cu ratio. Compared with the control group, MCI patients had significantly lower levels of plasma zinc and Zn/Cu (*p* < 0.001), while copper levels were significantly higher (*p* = 0.001).

**Table 1:** Demographics and clinical characteristics of participants.

Characteristics	Control (*n* = 136)	MCI (*n* = 136)	*t/Z/Χ* ^2^	*p*-value
Age (mean, SD)^a^	65.72 (6.73)	66.51 (7.07)	−0.95	0.252
MMSE score^a^	28.18 (0.87)	24.15 (2.75)	16.32	<0.001
Diabetes duration (years)^b^	6.00 (2.00–11.00)	6.00 (2.00–12.00)	−0.107	0.457
Plasma zinc (μg/L)^b^	936.29 (827.41–1205.58)	831.31 (717.91–1043.41)	3.573	<0.001
Plasma copper (μg/L)^b^	860.47 (750.25–974.34)	932.07 (808.73–1032.12)	−3.240	0.001
Plasma Zn/Cu ratio^b^	1.11 (0.91–1.34)	0.91 (0.75–1.17)	4.280	<0.001
Aβ40 (ng/L)^b^	207.61 (139.84–265.79)	173.95 (83.82–253.67)	2.361	0.009
Aβ42 (ng/L) ^b^	69.15 (56.03–85.54)	67.27 (54.18–88.19)	−0.330	0.371
Aβ42/40^b^	0.36 (0.24–0.52)	0.45 (0.27–0.90)	−2.415	0.008
Sex (male), *n* (%)^c^	51 (37.50)	51 (37.50)	0.000	1.000
Current smoker, *n* (%)^c^	28 (24.35)	39 (32.23)	1.803	0.196
Current drinker, *n* (%)^c^	23 (20.00)	21 (17.36)	0.272	0.620
Family history of diabetes, *n* (%)^c^	55 (40.44)	37 (27.21)	5.322	0.029
CHD, *n* (%)^c^	20 (14.71)	17 (12.50)	0.282	0.724
Hypertension, *n* (%)^c^	75 (55.15)	82 (60.29)	0.738	0.462
Hyperlipidemia, *n* (%)^c^	69 (50.74)	39 (28.68)	13.821	<0.001
APOE ε4 carrier, *n* (%)^c^	19 (13.97)	34 (25.00)	5.273	0.032

APOE, Apolipoprotein E; Aβ, β-Amyloid; Aβ42/40, the ratio of Aβ42 to Aβ40; CHD, coronary atherosclerotic heart disease; MMSE, mini-mental state examination; SD, standard deviation; Zn/Cu, the ratio of zinc to copper.

Data are presented as mean (SD) for parametrically distributed data, median (interquartile range [IQR]) for nonparametrically distributed data, and *n* (%) for categorical data.

^aStudent’s t-test.^

^bMann–Whitney U rank sum test.^

^cchi-square test.^

Plasma Aβ40 levels were significantly lower, while Aβ42/40 ratio was higher in MCI compared with controls. The median (interquartile range) plasma Aβ40 levels in MCI and control groups were 173.95 (83.82–253.67) ng/L and 207.61 (139.84–266.17) ng/L, respectively; plasma Aβ42 levels were 67.27 (54.18–88.19) ng/L and 69.15 (55.37–85.61) ng/L; and plasma Aβ42/40 ratio levels were 0.45 (0.27–0.90) and 0.36 (0.24–0.52).

### 3.2 Association of plasma zinc and copper levels with the risk of MCI in patients with T2DM

**Figure 1:**
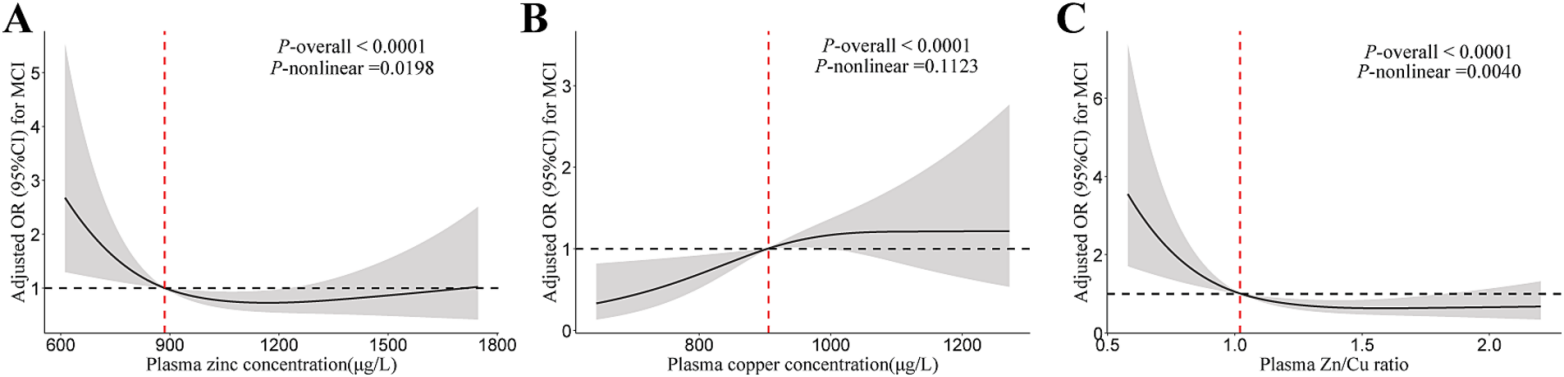
The restricted cubic spline for the association of plasma zinc, copper, and Cu/Zn ratio with MCI. **(A)** Plasma zinc; **(B)** Plasma copper; **(C)** Zn/Cu ratio. Results were adjusted for age, sex, diabetes duration, APOE ε4 carrier status, BMI, current smoking status, current drinking status, family history of diabetes, hypertension, hyperlipidemia, HbA1c, and CHD. Model for plasma zinc was additionally adjusted for plasma copper. Model for plasma copper was additionally adjusted for plasma zinc. MCI, mild cognitive impairment; Zn/Cu, the ratio of zinc to copper.

The plasma zinc level was non-linearly associated with lower MCI risk in T2DM patients (Figure 1). Compared to the lowest tertile, the OR (95% CI) for MCI were 0.28 (0.12, 0.64) and 0.33 (0.13, 0.79) for participants in the middle and highest tertiles of plasma zinc level, respectively (Table 2). For copper, a linear association between plasma copper and MCI risk was observed in T2DM patients (Figure 1). Compared with those in the lowest tertile of copper levels, participants in the middle and highest tertiles had 182 and 256% higher risk of MCI (adjusted OR [95% CI]: 2.82 [1.08, 7.38], 3.56 [1.42, 8.94], *p* for trend = 0.022), respectively. In addition, the Zn/Cu ratio was significantly associated with lower MCI risk in T2DM patients in a non-linear manner (Figure 1). Compared with those in the lowest tertile of Zn/Cu ratio, the multivariable-adjusted OR (95% CI) for MCI in the highest tertile was 0.37 (0.15, 0.93).

**Table 2:** Odds ratio of plasma zinc and copper concentrations with risk of MCI among individuals with T2DM^a^.

Variable	Tertiles of plasma metal concentrations			*P* for trend^b^	1-SD increment
	T1 (Lowest)	T2	T3 (Highest)		
Plasma zinc					
Range (μg/L)	< 867.61	867.61–1074.97	≥ 1074.97		
Median (μg/L)	739.72	963.15	1338.22		
Case/control, *n*	79/45	28/46	29/45		
Crude model	1.00 (reference)	0.28 (0.14, 0.57)	0.30 (0.15, 0.61)	0.004	0.65 (0.48, 0.88)
Model 1^c^	1.00 (reference)	0.31 (0.15, 0.65)	0.29 (0.14, 0.61)	0.020	0.65 (0.48, 0.90)
Model 2^d^	1.00 (reference)	0.28 (0.12, 0.64)	0.33 (0.13, 0.79)	0.019	0.71 (0.48, 1.05)
Plasma copper					
Range (μg/L)	< 780.80	780.80–931.19	≥ 931.19		
Median (μg/L)	736.60	855.93	1038.59		
Case/control, *n*	24/46	44/44	68/46		
Crude model	1.00 (reference)	2.68 (1.23, 5.85)	3.84 (1.80, 8.16)	0.039	1.60 (1.19, 2.17)
Model 1^c^	1.00 (reference)	2.49 (1.09, 5.72)	3.72 (1.67, 8.28)	0.043	1.60 (1.17, 2.20)
Model 2^d^	1.00 (reference)	2.82 (1.08, 7.38)	3.56 (1.42, 8.94)	0.022	1.47 (1.03, 2.11)
Plasma Zn/Cu ratio					
Range	<0.98	0.98–1.29	≥1.29		
Median	0.78	1.11	1.60		
Case/control, *n*	79/46	30/45	27/45		
Crude model	1.00 (reference)	0.44 (0.25, 0.78)	0.29 (0.14, 0.58)	0.056	0.50 (0.33, 0.74)
Model 1^c^	1.00 (reference)	0.44 (0.25, 0.78)	0.27 (0.13, 0.56)	0.049	0.48 (0.31, 0.74)
Model 2^d^	1.00 (reference)	0.48 (0.24, 0.95)	0.37 (0.15, 0.93)	0.107	0.56 (0.35, 0.91)

MCI, mild cognitive impairment; SD, standard deviation. T2DM, type 2 diabetes mellitus; Zn/Cu, the ratio of zinc to copper.

^a^Data are ORs (95% CIs).

^bLinear trend test using the median value of each category.^

^cModel 1 was adjusted for age, diabetes duration, and APOE ε4 carrier status.^

^dModel 2 was additionally adjusted for BMI, current smoking status, current drinking status, family history of diabetes, hypertension, hyperlipidemia, HbA1c, and CHD. Model 1, 2 for plasma zinc was additionally adjusted for plasma copper. Model 1, 2 for plasma copper was additionally adjusted for plasma zinc.^

### 3.3 Association of plasma zinc and copper levels with MMSE scores

**Figure 2:**
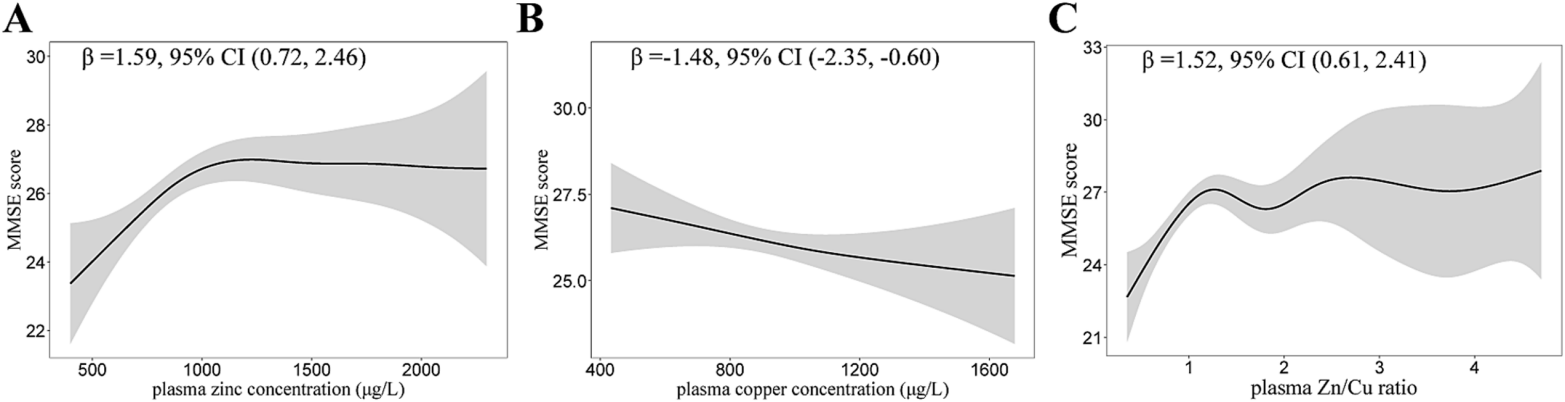
Generalized Additive Models (GAMs) for the association of plasma zinc, copper, and Zn/Cu ratio with MMSE scores. **(A)** Plasma zinc; **(B)** Plasma copper; **(C)** Zn/Cu ratio. Adjusted for age, diabetes duration, APOE ε4 carrier status, BMI, current smoking status, current drinking status, family history of diabetes, hypertension, hyperlipidemia, HbA1c, and CHD. Model for plasma zinc was additionally adjusted for plasma copper. Model for plasma copper was additionally adjusted for plasma zinc. MMSE, mini-mental state examination; Zn/Cu, the ratio of zinc to copper.

The dose–response relationship between plasma zinc, copper levels and MMSE scores are shown in Table 3. The higher plasma zinc was non-linearly associated with a higher MMSE score in T2DM patients (Figure 2). In contrast, the association between plasma copper levels and MMSE scores was negative. The relationship curve of Zn/Cu ratio showed an increasing trend; as the Zn/Cu ratio increases, the MMSE score also continue to increase.

**Table 3:** Generalized additive models for the association of plasma zinc, copper, and the Zn/Cu ratio with MMSE scores^a^.

Variable	Tertiles of plasma metal concentrations			*P* for trend^b^	1-SD increment
	T1	T2	T3		
Plasma zinc					
Range (μg/L)	< 867.61	867.61–1074.97	≥ 1074.97		
Median (μg/L)	739.72	963.15	1338.22		
Case/control, *n*	79/45	28/46	29/45		
Crude model	0.00 (reference)	1.44 (0.64, 2.23)	1.41 (0.61, 2.21)	0.0006	0.55 (0.21, 0.89)
Model 1^c^	0.00 (reference)	1.38 (0.55, 2.21)	1.36 (0.55, 2.18)	0.0024	0.47 (0.12, 0.82)
Model 2^d^	0.00 (reference)	1.56 (0.72, 2.40)	1.57 (0.70, 2.44)	0.0002	0.51 (0.15, 0.88)
Plasma copper					
Range (μg/L)	< 780.80	780.80–931.19	≥ 931.19		
Median (μg/L)	736.60	855.93	1038.59		
Case/control, *n*	24/46	44/44	68/46		
Crude model	0.00 (reference)	−0.90 (−1.77, −0.03)	−1.75 (−2.58, −0.92)	0.0142	−0.51 (−0.84, −0.17)
Model 1^c^	0.00 (reference)	−0.79 (−1.66, 0.07)	−1.55 (−2.39, −0.72)	0.0195	−0.43 (−0.77, −0.10)
Model 2^d^	0.00 (reference)	−0.89 (−1.79, 0.01)	−1.49 (−2.36, −0.62)	0.0072	−0.37 (−0.72, −0.02)
Plasma Zn/Cu ratio					
Range	< 0.98	0.98–1.29	≥ 1.29		
Median	0.78	1.11	1.60		
Case/control, *n*	79/46	30/45	27/45		
Crude model	0.00 (reference)	1.57 (0.78, 2.37)	1.45 (0.65, 2.25)	0.0001	0.62 (0.27, 0.98)
Model 1^c^	0.00 (reference)	1.62 (0.82, 2.41)	1.52 (0.71, 2.32)	< 0.0001	0.64 (0.28, 1.00)
Model 2^d^	0.00 (reference)	1.56 (0.73, 2.38)	1.52 (0.62, 2.42)	0.0002	0.62 (0.23, 1.00)

MMSE, mini-mental state examination; SD, standard deviation; Zn/Cu, the ratio of zinc to copper.

^a^Data are Betas (95% CIs).

^bLinear trend test using the median value of each category.^

^cModel 1 was adjusted for age, diabetes duration, and APOE ε4 carrying status.^

^dModel 2 was additionally adjusted for BMI, current smoking status, current drinking status, family history of diabetes, hypertension, hyperlipidemia, HbA1c, and CHD. Model 1, 2 for plasma zinc was additionally adjusted for plasma copper. Model 1, 2 for plasma copper was additionally adjusted for plasma zinc.^

### 3.4 Association of plasma zinc and copper levels with Aβ40 and Aβ42

**Figure 3:**
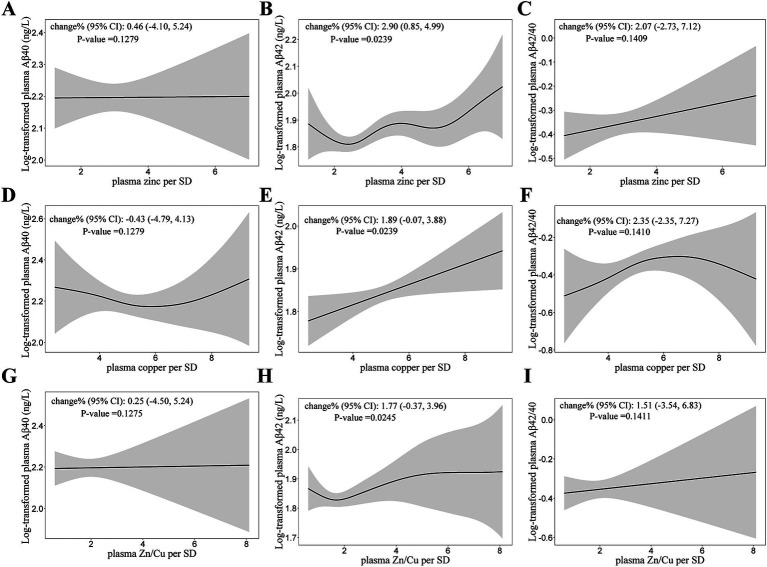
Generalized Additive Models (GAMs) for the association of log-transformed A*β* with plasma zinc, copper concentrations, and Zn/Cu ratio. **(A,D,G)** GAMs for the association of log-transformed Aβ40 with zinc, copper and Zn/Cu ratio; **(B,E,H)** GAMs for the association of log-transformed Aβ42 with zinc, copper and Zn/Cu ratio; **(C,F,I)** GAMs for the association of log-transformed Aβ42/40 with zinc, copper and Zn/Cu ratio. Adjusted for age, diabetes duration, APOE ε4 carrier status, BMI, current smoking status, current drinking status, family history of diabetes, hypertension, hyperlipidemia, HbA1c, and CHD. Model for plasma zinc was additionally adjusted for plasma copper. Model for plasma copper was additionally adjusted for plasma zinc. Aβ, β-Amyloid; Aβ42/40, the ratio of Aβ42 to Aβ40; Zn/Cu, the ratio of zinc to copper.

The relationship of plasma zinc and copper levels with Aβ40 and Aβ42 are shown in Table 4. Plasma zinc and copper concentration were both positively associated with Aβ42 (Figure 3). For per SD (327.71 μg/L) increase in plasma zinc levels, the percent change (95% CI) of Aβ42 was 2.90 (0.85, 4.99). Plasma zinc and copper levels were not significantly associated with Aβ40 nor Aβ42/40.

**Table 4:** Association of plasma zinc and copper with plasma Aβ40 and Aβ42.

Variables	Aβ40 [Change% (95% CI)]^a^	*p* value	Aβ42 [Change% (95% CI)]	*p* value	Aβ42/40 [Change% (95% CI)]	*p* value
Plasma zinc (per SD)						
Crude model	−1.07 (−5.19, 3.21)	0.1272	2.09 (0.11, 4.10)	0.0269	3.23 (−1.25, 7.91)	0.1388
Model 1^b^	−1.12 (−5.32, 3.27)	0.1285	2.61 (0.60, 4.67)	0.0267	3.16 (−1.34, 7.86)	0.1400
Model 2^c^	0.46 (−4.10, 5.24)	0.1279	2.90 (0.85, 4.99)	0.0239	2.07 (−2.73, 7.12)	0.1409
Plasma copper (per SD)						
Crude model	−0.32 (−4.43, 3.98)	0.1274	2.00 (0.04, 3.99)	0.0270	2.34 (−2.08, 6.94)	0.1393
Model 1^b^	−0.79 (−4.96, 3.58)	0.1285	2.48 (0.49, 4.52)	0.0267	3.30 (−1.22, 8.05)	0.1394
Model 2^c^	−0.43 (−4.79, 4.13)	0.1279	1.89 (0.00, 3.88)	0.0239	2.35 (−2.35, 7.27)	0.1410
Plasma Zn/Cu ratio (per SD)						
Crude model	−0.95 (−5.34, 3.66)	0.1273	1.07 (−1.03, 3.21)	0.0273	2.04 (−2.69, 7.00)	0.1394
Model 1^b^	−0.75 (−5.18, 3.89)	0.1281	1.11 (−1.00, 3.27)	0.0275	1.89 (−2.88, 6.88)	0.1406
Model 2^c^	0.25 (−4.50, 5.24)	0.1275	1.77 (−0.37, 3.96)	0.0245	1.51 (−3.54, 6.83)	0.1411

Aβ, β-Amyloid; Aβ42/40, the ratio of Aβ42 to Aβ40; SD, standard deviation; Zn/Cu, the ratio of zinc to copper.

^a^Percent change ((exp(beta) − 1) × 100) (95% CI) was calculated by Generalized additive models.

^bModel 1 was adjusted for age, sex, diabetes duration, and APOE ε4 carrying status.^

^cModel 2 was additionally adjusted for BMI, current smoking status, current drinking status, family history of diabetes, hypertension, hyperlipidemia, CHD and HbA1c. Model 1, 2 for plasma zinc was additionally adjusted for plasma copper. Model 1, 2 for plasma Cu was additionally adjusted for plasma zinc.^

## 4 Discussion

In this age- and sex-matched case–control study, plasma zinc concentration was significantly associated with lower MCI risk in T2DM patients after adjusting for multivariable confounders. In addition, higher plasma copper concentration was associated with higher MCI risk, while higher zinc-to-copper ratio was significantly associated with lower MCI risk in T2DM patients.

In this study, the median plasma zinc concentrations in the MCI group and control group were 831.31 (717.91–1043.41) μg/L and 936.29 (827.41–1205.58) μg/L, respectively. These findings are consistent with previous reports (27, 38–39). Our findings confirm previous studies linking low plasma zinc levels with a heightened risk of cognitive decline and neurodegenerative diseases. Specifically, the lower zinc levels in MCI patients compared to controls may indicate a diminished neuroprotective effect, potentially exacerbating cognitive dysfunction in T2DM patients. Conversely, plasma copper levels were found to be significantly higher in MCI patients. This finding is consistent with prior research suggesting that elevated copper concentrations can contribute to the formation of reactive oxygen species and oxidative stress, both of which have been implicated in the pathophysiology of neurodegenerative diseases, including AD (40). Elevated copper levels may also interfere with the balance of metal ions in the brain, exacerbating neuronal damage and accelerating cognitive decline. Furthermore, our findings indicate that the risk of MCI increases with higher copper levels corroborates the hypothesis that dysregulated metal homeostasis could be a key factor in the development of cognitive dysfunction in T2DM patients (19). Another key variable Zn/Cu ratio examined in this study was found to be inversely associated with MCI risk. This finding supports the notion that maintaining an optimal balance between zinc and copper is crucial for cognitive health (17). A dysregulated Zn/Cu ratio could impair cellular antioxidant defense, further increasing the risk of neurodegeneration. Previous studies have shown that the Zn/Cu ratio serves as an indicator of systemic metal balance, which is critical for maintaining brain health and preventing cognitive decline (41). The risk of MCI in T2DM patients significantly decreased with increasing zinc concentration and Zn/Cu ratio, and decreasing copper concentration could decrease the risk.

Additionally, we also examined the relationship between plasma zinc and copper levels and plasma Aβ peptides. In our study, plasma Aβ40 levels were lower in MCI compared with controls, while the plasma Aβ42/40 ratio was higher in MCI cases. These findings parallel with previous study, which identified an increased plasma Aβ42- to- Aβ40 ratio as an independent risk factor for cognitive decline in T2DM patients (8). The disruption of Aβ metabolism in both the brain and the periphery has been identified as the underlying mechanism linking the development of diabetes with AD patients (23). A study estimated that in the human central nerve system, the fractional production rate of Aβ is 7.6% per hour, while the clearance rate is 8.3% per hour, respectively (42). The preclinical stage of AD has been identified by the abnormal levels of cerebrospinal fluid (CSF), abnormal blood biomarkers including Aβ, tau, and neurofilament light chain, and the presence of normal cognitive function (43). However, changes of CSF or plasma Aβ levels observed were contradictory in AD and control individuals. Some studies reported that the increase of plasma Aβ42 or the Aβ42/40 ratio may be risk factors for AD, while others reported a negative association (35). The differences in these findings may due to most studies being cross-sectional and regarding biomarkers in sporadic AD, failing to capture biomarkers changes during the progression from normal cognitive to AD (43).

To further investigate the potential role of plasma zinc deficiency as a critical factor and intervention target in impaired peripheral Aβ clearance and cognitive dysfunction in individuals with T2DM, this study also examined the association between plasma metals and Aβ40, Aβ42 in T2DM patients. Our findings revealed that the plasma zinc and copper levels were positively associated with Aβ42 but showed no significant association with Aβ40 or Aβ42/40. This lack of association may due to the Aβ deposition in the brain, which subsequently reduces plasma amyloid beta levels (44). On the contrary, a previous study about blood biomarker and cognitive impairment showed plasma zinc levels were negatively correlated with plasma Aβ42 levels (45). These results suggest that while zinc and copper may influence amyloid metabolism, their effects may be more pronounced in specific forms of amyloid beta, such as Aβ42, which is more closely linked to neurodegenerative processes in AD.

There are several strengths in our study. Firstly, we conducted a case-control study in T2DM patients, matching cases and controls by age and sex to minimize the potential confounding effects. Secondly, we explore the association of plasma zinc and copper levels with MCI in T2DM patients to identify potential risk factors. Moreover, the sensitive and reliable ICP-MS method was used to objectively measure plasma zinc and copper levels.

This study had several limitations. First, the retrospective and observational design prevented the evaluation of a causal relationship between plasma zinc, copper, the Zn/Cu ratio and MCI in T2DM patients. Second, the maintenance of zinc homeostasis is dependent on the dynamic equilibrium, and the factors unrelated to zinc status or dietary zinc intake, including tissue catabolism, infection, inflammation, or medications can change the zinc concentration. Third, the limitation of this study was the inability to evaluate the utility of other biomarkers, such as urinary and other tissue zinc and copper levels, in assessing zinc and copper status. Zinc and copper concentrations are known to be affected by other factors, such as inflammation, and fasting or postprandial states. Nevertheless, blood zinc and copper concentrations are currently regarded as valuable and reliable biomarkers.

The investigation confirms a significant association between plasma zinc and copper concentrations and the prevalence of MCI in patients with T2DM.Furthermore, our study provides insights into the relationships between plasma zinc, copper, the Zn/Cu ratio, and Aβ levels in MCI, highlighting their potential role as biomarkers for T2DM-related cognitive impairments. These findings underscore the importance of trace element homeostasis in cognitive function and may inform the development of novel therapeutic strategies aimed at preventing or mitigating cognitive decline in T2DM patients.

## Data Availability

The raw data supporting the conclusions of this article will be made available by the authors, without undue reservation.
